# Genome Structures and Evolution Analysis of *Hsp90* Gene Family in *Brassica napus* Reveal the Possible Roles of Members in Response to Salt Stress and the Infection of *Sclerotinia sclerotiorum*

**DOI:** 10.3389/fpls.2022.854034

**Published:** 2022-04-07

**Authors:** Long Wang, Fei Liu, Lingyue Ju, Bing Xue, Yongfeng Wang, Daojie Wang, Dianyun Hou

**Affiliations:** ^1^College of Agriculture, Henan University of Science and Technology, Luoyang, China; ^2^State Key Laboratory of Cotton Biology, School of Life Sciences, Henan University, Kaifeng, China; ^3^College of Agriculture, Henan University, Kaifeng, China

**Keywords:** *Brassica napus*, heat shock protein, Hsp90, salt stress, *Sclerotinia sclerotiorum*

## Abstract

Heat shock proteins 90 (Hsp90s) are conserved proteins participating in the responses to heat stress and are found to be involved in different kinds of abiotic and biotic stresses. *Brassica napus* (*B. napus*) is an important heteropolyploid crop, producing edible oil. Salt stress is one of the most important hazards to the growth of rape in the world, while Sclerotinia stem rot is one of the most serious diseases, caused by *Sclerotinia sclerotiorum* (*S. sclerotiorum*). In this study, the evolution of *Hsp90* genes and their responses to these two stresses were elucidated. Bioinformatic analysis through the whole genome of *B. napus* identified 35 *Hsp90* gene family members. Five groups were obtained *via* phylogenetic analysis with the 35 *Hsp* genes, *Hsps* from its two ancestor species *Brassica rapa*, *Brassica oleracea*, and *AtHsps*. Gene structure and conservative motif analysis of these 35 *Hsps* indicated that the *Hsps* were relatively conservative in each group. Strong collinearity was also detected between the genomes of *Brassica rapa*, *Brassica oleracea* and *B. napus*, along with identifying syntenic gene pairs of *Hsps* among the three genomes. In addition, whole genome duplication was discovered as the main reason for the generation of *BnHsp* gene family. The analysis of cis-acting elements indicated that *BnHsp90* might be involved in a variety of abiotic and biotic stress responses. Analysis of the expression pattern indicated that *BnHsp90* participates in the responses of *B. napus* to salt stress and the infection of *S. sclerotiorum*. Fourteen and nine *BnHsp90s* were validated to be involved in the defense responses of *B. napus* against salt stress and *S. sclerotiorum*, respectively. Our results provide new insights for the roles of *BnHsp90s* in the responses of *B. napus* to salt stress and *S. sclerotiorum*.

## Introduction

Oilseed rape (*Brassica napus* L, *B. napus*) belongs to the cruciferous family and is one of the most widely cultivated oil crops in the world. As it is the third most-used crop for oil production, rapeseed has important economic value in the world’s oil crops (Gupta, 2016). Seeds of rapeseed are rich in fat (40–49%) and suitable for producing edible oil. It can also be used for animal feed for its high protein content as well as industrial purposes, such as high-quality lubricant (Warner and Lewis, 2019). *B. napus* is a heteropolyploid species formed by natural hybridization. It is formed by the hybridization of *Brassica rapa* (*B. rapa*, AA = 20) and *Brassica oleracea* (*B. oleracea*, CC = 18), two diploid species (Chalhoub et al., 2014).

Plants often suffer from biotic and abiotic stresses, which have a great impact on the growth of plants. Biological stress and abiotic stress are normally interrelated and together affect the growth of plants (Chaves et al., 2003). In addition, high temperature, cold, drought, salt, nutrient deficiency, heavy metal toxicity, strong light, ozone and other abiotic stresses have important effects on the yield and quality of crop plants (Yamaguchi-Shinozaki and Shinozaki, 2006; Sham et al., 2014, 2015, 2019). As we know, in recent decades global warming has become an important factor that promotes the occurrence of extreme weather. Extreme high temperatures have become common, intensifying the abiotic and biotic stresses, affecting plant growth and crop production (Teshome et al., 2020).

In the process of long-term evolution, plants gradually formed a set of regulatory mechanisms to respond to changes in environmental factors (Cheng et al., 2019). When plants are subjected to heat stress or other stress stimuli, they will produce some highly conserved stress proteins, which are called heat shock proteins (Hsps; Queitsch et al., 2002). According to the molecular weight, heat shock proteins are divided into five categories, Heat shock protein (Hsp) 90 (Hsp90), Hsp70, Hsp60 and small heat shock protein (Pearl and Prodromou, 2000). Hsp90 is highly conserved among heat shock proteins which exist widely in eukaryotic cells, functioning as a molecular chaperone (Pearl and Prodromou, 2000). Previous studies have shown that the *Hsp90* gene is involved in the folding of transcription factors and kinases, stressing the conduction of stress signals (Wegele et al., 2004). The core structure of *Hsp90* contains the N-terminal ATP binding domain, the middle domain and the C-terminal dimerization domain (Pearl and Prodromou, 2000). In animal cells, Hsp90 functions mainly in the cytoplasm, mainly as Hsp90-α and Hsp90-β (Krishna and Gloor, 2001). In recent years, a large number of *Hsp90* genes have been discovered in many plants (Di Donato and Geisler, 2019). Seven *Hsp90* genes were identified in *Arabidopsis* (Krishna and Gloor, 2001). In plants, apart from cytoplasmic Hsp90, endoplasmic reticulum and plastid-localized Hsp90s were also identified (Schröder et al., 1993).

Recent studies have shown that *Hsp90* affects the resistance of plants to biotic stresses and abiotic stresses (Di Donato and Geisler, 2019). Hsp90 protein accumulation was found to be altered in cold-treated winter wheats followed cold treatment (Vitamvas et al., 2012). In other plants, *Hsp* genes were also found to be induced by or participate as an important role in other stresses, such as cold (Krishna et al., 1995), pathogens (McLellan et al., 2007; Moshe et al., 2016), oxidative stress (Nishizawa-Yokoi et al., 2010; Sable et al., 2018), drought (Song et al., 2009). Overexpression of *AtHsp90.2*, *AtHsp90.5* and *AtHsp90.7* in *A. thaliana* can enhance plant sensitivity to salt and drought stress (Song et al., 2009). Under stress conditions, heat shock transcription factor (HSF) binds to the heat shock element (HSE) in the *Hsp90* gene promoter to initiate the transcription of the *Hsp90* gene (Lohmann et al., 2004). Apple Hsp90, a chaperone protein, interacts with MdHSFA8a to inhibit its binding activity and transcriptional activation, while the complex of MdHSP90-MdHSFA8a dissociates and releases MdHSFA8a to promote the accumulation of flavonoids, removes active oxygen, and increases the survival ability of plants under drought conditions (Wang et al., 2020). In tomato, the transcript level of *Hsp90* increased under high temperature and compound stress, but decreased under drought stress (Raja et al., 2020). In *B. napus*, expression of *Hsp90* is up-regulated under cold stress conditions or chromium stress (Krishna et al., 1995; Gill et al., 2015). As a plasma-membrane associated cation-binding protein, Hsp90 was indicated as a key role in the drought resistance of *B. napus via* comparative proteome analysis (Mohammadi et al., 2012).

During the growth of oilseed rape, the threats encountered include sclerotinia stem rot, which is one of the most serious diseases on oilseed rape. The causal agent of this disease is *Sclerotinia sclerotiorum* (*S. sclerotiorum*), which results in enormous economic losses around the world (Bolton et al., 2006). Salicylic acid, an important phytohormone, can activate plants’ resistance to biotrophic and hemibiotrophic pathogens and establishes systemic resistance, while the combination of JA and ET signals is responsible for resistance activation to necrotrophic pathogens (Glazebrook, 2005; Mengiste et al., 2009; Abuqamar et al., 2017). *Hsp90* genes were found to be involved in the defense responses of plants to different pathogens through regulating SA levels (Makarova et al., 2018; Yang et al., 2019; Wei et al., 2021). In the interaction between *B. napus* and *S. sclerotiorum*, salicylic acid was also discovered as an important defense signal, and Hsp90s might contribute to *B. napus* resistance to *S. sclerotiorum* as well (Yang et al., 2018; Nie et al., 2019).

In *Arabidopsis*, seven *Hsp90* genes have been found and some of them had been reported to be involved in the modulation of plant responses to biotic stresses (Schopf et al., 2017). However, the identification of the *Hsp90* gene family in the closely related species, *B. napus*, has not yet been completed. In this study, we identified and elucidated the gene structure, conserved domains of *Hsp90* gene family, as well as the evolutionary relationship of these members between *B. napus* and its closely related species *A. thaliana*, *B. rapa*, and *B. oleracea*. In addition, the transcriptional responses of *BnHsp90* gene family to salt stress and the infection of *S. sclerotinia* were investigated.

## Materials and Methods

### Identification of *Hsp90* Gene Family Members From *B. napus*, *B. rapa*, and *B. oleracea*

In order to identify *Hsp90* genes, the sequences of seven AtHsp90 proteins were retrieved from the *A. thaliana* genome,^1^ and were subsequently used for determining the *Hsp90* genes in the genomes of *B. napus* (v5),^2^ *B. rapa* (Brara_Chiifu_V3.0)^3^ and *B. oleracea* (Brara_Chiifu_V3.0, Braol_JZS_V2.0; see footnote 3) *via* reciprocal blast with BLASTP program (Altschul et al., 1997). The default parameters with E-value less than 1E-10 were set in the BLASTP searches.

### Phylogenetic Analysis of *Hsp90* Gene Family

The Hsp90 protein sequences of *B. napus*, *B. rapa* and *B. oleracea* and *A. thaliana* were merged and performed multiple sequence alignment using ClustalW program (Larkin et al., 2007). Phylogenetic relationships of *Hsp90* genes were constructed with alignment results through FastTree software (Price et al., 2009).

### Chromosomal Mapping, Duplicated Type, and Collinear Blocks Analysis

Chromosomal position information for *BnHsp90* genes was extracted from generic feature format (GFF) files which were downloaded from *B. napus* genome website (see footnote 2). The positions of *BnHsp90* genes were indicated on the corresponding chromosomes with TBtools software (Chen et al., 2020). MCscanX software (Wang et al., 2012) was used to identify the gene duplication types and collinearity relationships. Self-self comparison was performed using BLASTP program between *B. napus* protein sequences with the e-value under 1e-10. Duplication types of all the *B. napus* whole genome proteins were identified using the program incorporated in MCScanX, and then duplication information of *BnHsp90* gene family was isolated. The collinear gene pairs within *BnHsp90* gene family were detected using the program detect_collinearity_within_gene_families.pl. of MCscanX, and the collinearity relationships of these gene pairs were displayed using Circos software (Krzywinski et al., 2009).

### Analysis of the Gene Structures and Protein Conserved Domains in *BnHsp90* Gene Family

The information of gene structures (exon/intron) for *BnHsp90* genes was separated from GFF files and displayed using TBtools software (Chen et al., 2020). Protein sequences of BnHsp90 were submitted to MEME website^4^ for conserved motif analysis, and the display of conserved motifs were conducted in TBtools software (Chen et al., 2020). The R library ggseqlogo (Wagih, 2017) was employed to generate the sequence logos of the five most detected conserved motifs.

### Cis-Elements Prediction in the Promoter of *BnHsp90* Genes

Promoter information of *BnHsp90* genes was extracted from GFF files of *B. napus*, and the promoter sequences were isolated using seqtk software (Li, 2013). A total of 2,000 bp upstream sequences of the coding region were obtained and submitted to PlantCare^5^ for cis-elements analysis.

### Expression Analysis of *BnHsp90* Genes in Response to Abiotic and Biotic Stresses Based on Published RNA-Seq Data

To investigate the transcriptional patterns of *BnHsp90* genes in response to salt and the infection of *S. sclerotiorum*, SRA files were downloaded from GEO RNA-seq datasets (GSE81545) and Sequence Read Archive (SRA) database (PRJNA561674). All reads were checked the quality and cleaned using fastp software (Chen et al., 2018). Gene expression quantification was performed using a software package called Kallisto (Bray et al., 2016). The transcripts per kilobase of exon model per million mapped reads (TPM) values were separated and heat maps were constructed from relative TPM value using pHeatmap software package (Raivo, 2012).

### Plant Materials and Stress Treatments

*B. napus* plants are grown in an incubator at 22°C ~ 25°C, with a day and night photoperiod of 16/8 h. Variety K407 was provided by the Hybrid Rapeseed Research Center of Shanxi Province, and was used for treatment. Seeds of K407 were sterilized in 30% bleach and germinated on one-layer nylon mesh with support below, floating onto the sterile water. Seven-day-old seedlings after germination were transferred into Hoagland solution with foams as supports. The Hoagland solution was replaced every week until the plants ready for treatment. Plants in four-leaf stage were moved to the Hoagland solution containing 150 mM NaCl or without NaCl. After processing, samples were taken at 0 h, 1 h, 3 h, and 6 h, respectively. The collected true leaves were quickly frozen in liquid nitrogen. *Sclerotinia sclerotiorum* strains (separated from the stubbles of oilseed rape in the fields of Kaifeng, Henan, designated as Hns-1) were cultured on potato dextrose agar (PDA) plates. The actively growing mycelium at the edge of the colony on the mycelium agar block was taken out and inoculated onto the leaves of *B. napus*. Similarly, as a mock inoculation control, the leaves were inoculated with blank PDA agar blocks. The leaves from the inoculation area were collected at 0 h, 12 h, 24 h, and 48 h after inoculation, and immediately frozen in liquid nitrogen. All samples were stored in the refrigerator at −80°C until RNA extraction. Three independent plants produce a biological replicate, and each treatment consists of three independent biological replicates.

The materials and corresponding treatments in RNA-seq analysis from publicly available data can be found in the reports by Tan et al. (2019) and Girard et al. (2017).

### RNA Extraction and Quantitative RT-PCR

RNAprep Pure Plant Plus Kit (TIANGEN, China) was used to extract total RNA. One microgram of total RNA was revers-transcribed into cDNA using HiScript II Q RT SuperMix (Vazyme, R223, Nanjing). Quantitative RT-PCR (qPCR) was performed using ChamQ Universal SYBR qPCR Master Mix (Vazyme, Nanjing). Relative transcript levels of *BnHsp90s* were analyzed by quantitative real-time PCR using actin gene (*BnaA10g22340D*) as an internal control. The PCR amplification was performed using LightCycler^®^480 (Roche Diagnostics, United States). All experiments were performed from three biologically independent RNA samples, and each qPCR test had three replicates. Gene-specific primers were designed with Primer Premier 5.0 (PREMIER Biosoft, USA). The detailed information on the primers is shown in Supplementary Table S1.

### Subcellular Localization of BnA03.Hsp90.2

Open reading frame of *BnA03.Hsp90.2* was cloned into pEarLeyGate103 to generate a green fluorescent protein (GFP)-tagged expression vector (103-BnA03.Hsp90.2). GFP under the control of 35S promoter was used as negative control (35S-GFP), and H2B fused with mCherry was used for positive control (H2B-mCherry). Agrobacterium containing corresponding plasmids was infiltrated into *Nicotiana benthamiana* leaves for transient expression of fusion protein. Forty-eight hours after infiltration, the fluorescence signal was observed using a confocal laser scanning microscope (Leica TCS SP8).

### Transcriptional Activation Assay of BnA03.Hsp90.2 in Yeast Cells

The complete coding sequence of *BnA03.Hsp90.2* was fused into the pGBKT7 vector to generate pGBKT7-BnA03.Hsp90.2 plasmid. The pGBKT7-BnA03.Hsp90.2, pGBKT7-AtDREB (positive control) or pGBKT7 empty vector (negative control) was transformed into the AH109 yeast strain separately. The obtained positive transformants were grown on SD/-Trp and SD/-Trp-His medium. The transcriptional activation of each transformant was assessed based on their survival after incubation at 30°C for 3 days. Primers used for constructing the plasmids were stored in Supplementary Table S1.

### Statistical Analysis

Expression levels were calculated using 2^−ΔΔCT^ method (Livak and Schmittgen, 2001). ANOVA analysis was employed to assay the difference between samples or treatments, with value of *p* less than 0.05 to indicate the significant differences. The office software Excel 2016 was used for data analysis. The expression levels were from three different samples or treatments. Values in the histogram were represented as average values ± standard errors.

## Results

### Identification of *Hsp90* Genes in *B. napus*

The sequences of seven *A. thaliana* Hsp90 proteins were used to identify the *Hsp90* family members in the *B. napus* genome *via* reciprocal BLASTP. A total of 35 candidate *Hsp90* genes were detected, and were designated with their orders on each chromosome (Supplementary Table S2). The number of *Hsp90s* allocated on each chromosome is different: chromosome A03 contains the greatest number (6) of *Hsp90* genes, chromosome C02 ranks the second, with 5 *Hsp90s* on it, and followed by chromosome C03, with 4 *Hsp90s* on it (Supplementary Figure S1). *BnHsp90* genes are not randomly distributed on each chromosome, and mostly distributed at both ends of chromosomes, with many genes clustered (Supplementary Figure S1; Supplementary Table S2). On the chrA03, chrC02 and chrC03 chromosome, there are four or five *Hsp90* genes distributed in clusters (Supplementary Figure S1; Supplementary Table S2). Overall, a total of 35 *BnHsp90* genes were identified, with some genes clustered on ends of chromosomes.

The number of amino acids and biophysical properties vary among BnHsp90 proteins. The lengths of BnHsp90 proteins range from 63 to 872 aa (Supplementary Table S2). There are 27 BnHsp90 proteins with more than 300 aa (Supplementary Table S2). Accordingly, the molecular weights of different Hsp90s differ greatly, with fluctuations ranging from 7016.9 to 100512.9 Da (Supplementary Table S2). The number of exons encoding proteins is between 2 and 21 (Supplementary Table S2). In addition, the theoretical isoelectric point is distributed between 3.8 and 9.2, with BnA10.Hsp90.2 having the highest isoelectric point, 9.2, showing slightly alkalinity (Supplementary Table S2). Besides BnA10.Hsp90.2, the isoelectric point of other BnHsp90 proteins falls between 3.84 and 5.99, which is slightly acidic (Supplementary Table S2). To provide useful clues to reveal the functions of BnHsp90s, the subcellular locations of 35 Hsp90s proteins were predicted, and they were indicated to be in different organelles: There are 7 proteins located in cytoplasmic, 21 proteins in mitochondrial, 3 proteins in nuclear, and 4 proteins in other organelles (Supplementary Table S2).

### Phylogenetic Analysis *Hsp90* Genes Among *B. napus* and Its Closely-Related Species

**Figure 1:**
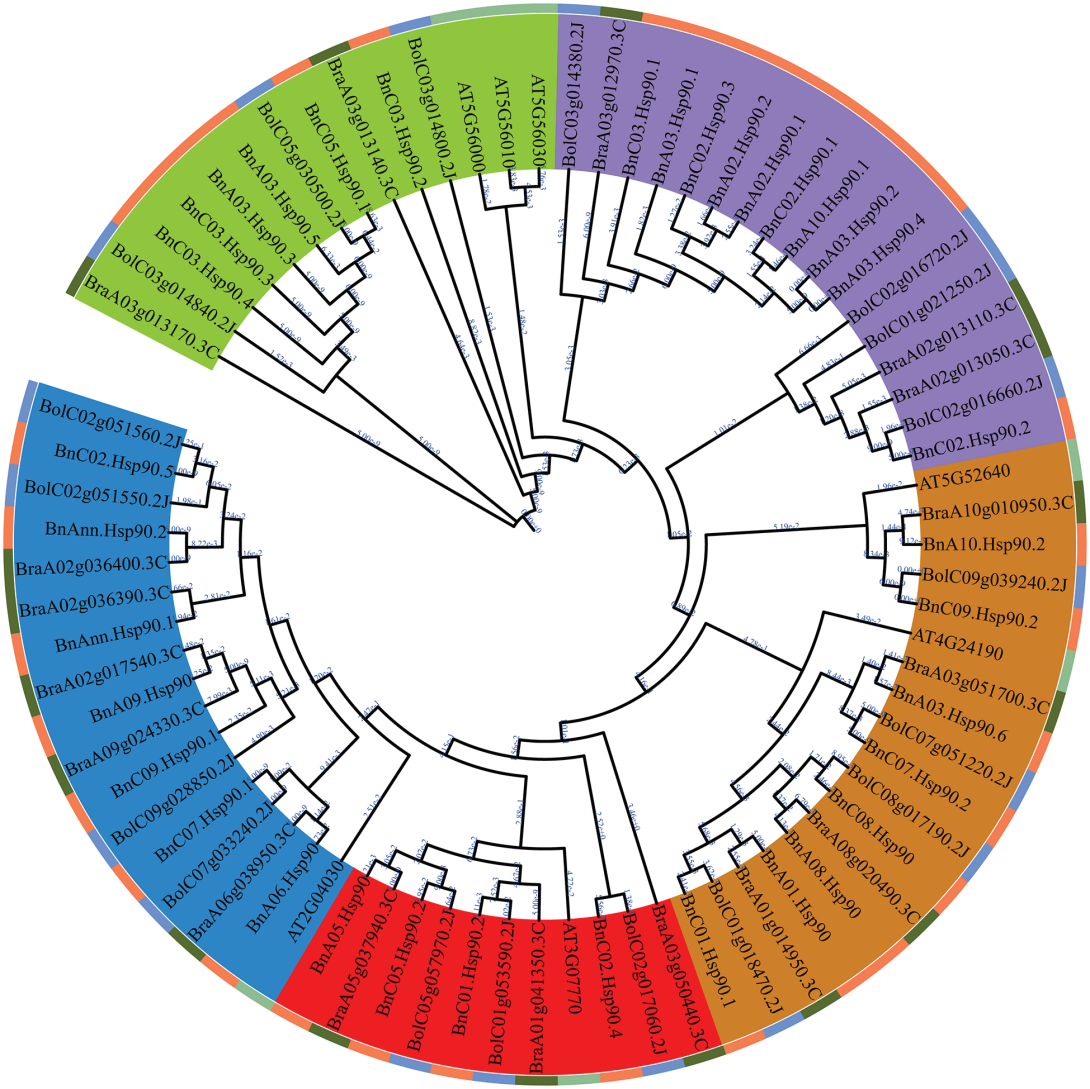
Phylogenetic tree of Hsp90 proteins from *A. thaliana*, *B. rapa*, *B. oleracea*, and *B. napus*. The Hsp90s were divided into five groups (groups I–V) based on the clustering of the protein sequence. The proteins from *A. thaliana*, *B. rape*, *B. oleracea* and *B. napus* are presented in light green, blue, dark green, and orange, respectively. The IDs of proteins from *Brassica napus*, *Brassica rapa*, *Brassica oleracea*, and *Arabidopsis thaliana* start with Bn, Bra, Bol, and AT, respectively. Branch length is indicated on each branch.

In order to further clarify the evolutionary relationship of the *Hsp90* gene family, FastTree software was used to construct a phylogenetic tree of Hsp90 proteins of *A. thaliana*, *B. rapa*, *B. oleracea* and *B. napus*. A phylogenetic tree was constructed using 7 AtHsp90s from *A. thaliana*, 17 BrHsp90s from *B. rapa*, 18 BoHsp90s from *B. oleracea* and 35 BnHsp90s from *B. napus*. According to the phylogenetic relationship of these proteins, Hsp90s were divided into five groups (groups I–V), containing 17, 11, 18, 17, and 14 proteins, respectively (Figure 1). The third group has the largest number of members, with 18. There is no Hsp90 from *Arabidopsis* in the fourth group. By analyzing the subcellular location, it was found that most of the genes in groups I, II, and III were located to mitochondria.

### Gene Structures and Conserved Motifs Among *BnHsp90* Gene Family

**Figure 2:**
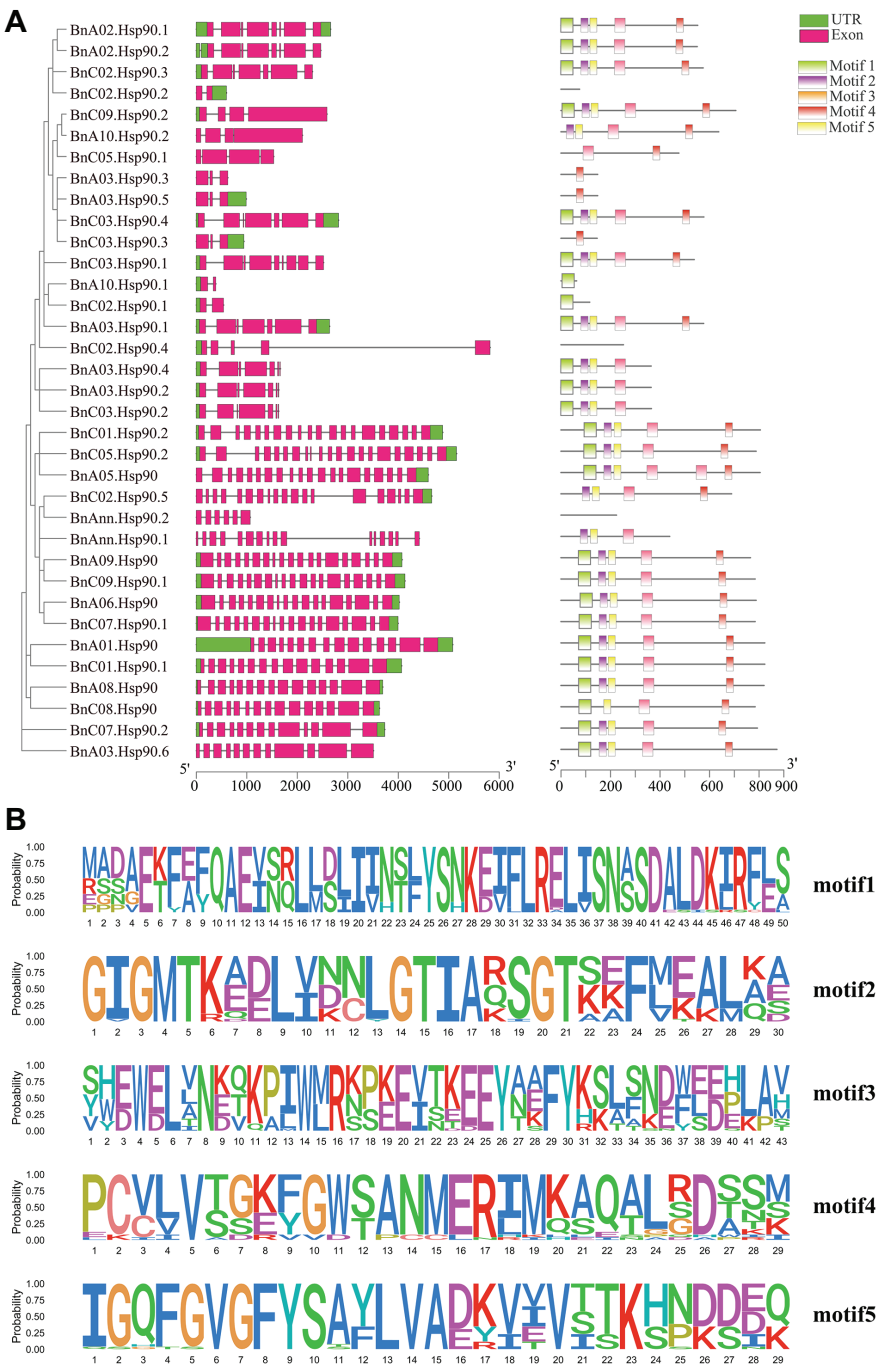
Gene structure and motif analysis of *BnHSP90s*. **(A)** left, Intron and exon structure of the *B. napus BnHsp90* genes according to the phylogenetic relationships. The untranslated region (UTR) and exon are indicated as green and pink boxes, respectively. The gray line represents introns. A, right, Distribution of the BnHsp90 conserved motifs in *B. napus*. There are five types of conserved motifs, which are represented by light green, purple, orange, red, and yellow. **(B)** MEME motifs are stacked by letters at each investigated site. The x-axis represents the width of the motif, whereas the y-axis represents the frequency of each letter.

The exon-intron structure analysis can provide important evidence support for the evolution of gene families. In order to analyze the exon-intron structure of *BnHsp90* coding region, the genome and coding sequences of *BnHsp90* were compared. The results showed that most members of *BnHsp90s* contained multiple exons, and the number of introns vary greatly (Supplementary Table S2; Figure 2A). As shown in Figure 2A; Supplementary Table S2, the gene structures are complicated; *BnA10.Hsp90.1*, *BnC02.Hsp90.1*, and *BnC02.Hsp90.2* contains two exons, but *BnC05.Hsp90.2* contains 21 exons (the greatest number; Supplementary Table S2).

We further analyzed the conserved motifs of *BnHsp90s* and determined the conservative pattern of amino acid residues in their domains. The most 5 conserved motifs detected in *BnHsp90* are indicated in Figure 2B, containing 29–43 amino acids. Motif 1 and 2 contain 30 amino acids, and Motif 3 and 4 contain 43 amino acids (Figure 2B). Among them, motif 5 has the lowest amino acid content, which is 29 amino acids. Among all *BnHsp90* members, 25 proteins contain motif 1, 25 proteins include motif 2, 27 proteins include motif 3, 26 proteins include motif 4 and 26 proteins include motif 5 (Figure 2B). Further analysis indicated that most of the motif 2, motif 4, motif 5 are more conservative. Similar to the intron patterns observed in each gene, the number of conserved motifs was relatively consistent in a single *Hsp90* gene paralog. Two-thirds of *Hsp90* genes contain motifs 1–5. Among them, some very conservative sequences were discovered, similar to the results in previous studies (Zhang et al., 2017).

### Gene Collinearity and Duplication Analysis of *BnHsp90* Genes

**Figure 3:**
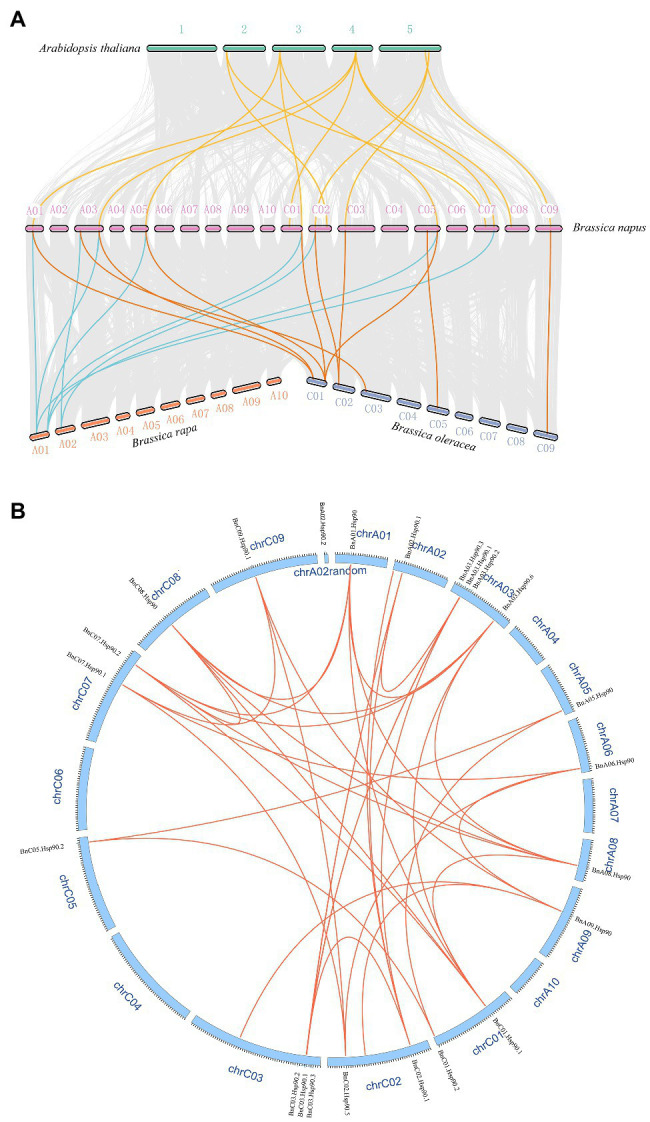
Syntenic relationships of *Hsp90s* among *A. thaliana*, *B. rapa*, *B. oleracea*, and *B. napus*. **(A)** *Hsp90* genes are plotted against their predicted counterparts in the four species. The chromosomes of *A. thaliana* are displayed as number 1–5, and the chromosomes of *B. napus* are showed in the form that symbol starting with A represents the chromosome originating from *B. rapa*, and symbol starting with C denotes the chromosome from *B. oleracea*. The chromosomes of *B. rapa* and *B. oleracea* are named using symbols beginning with A and C, respectively. The grey lines indicate the collinear blocks between different species. The yellow lines represent the collinear Hsp90 gene pairs between *A. thaliana* and *B. napus*. The blue lines denote the collinear Hsp90 gene pairs between *B. napus* and *B. rapa*, and the orange lines display the collinear gene pairs between *B. napus* and *B. oleracea*. **(B)** The collinear gene pairs of *BnHsp90s* on *B. napus* chromosomes. Red lines were used to link a pair of collinear genes. The ID of each chromosome was represented using “chr” followed by its ID, and the name of each gene was indicated on the corresponding chromosome.

A syntenic analysis was performed between *A. thaliana*, *B. napus* and its ancestors to speculate the evolutionary origin of *Hsp90* genes. The results indicated that many chromosomal rearrangements or gene duplications exist between *B. napus* and *B. rapa* and *B. oleracea*, and strong collinearity was also detected between them (Figures 3A,B). Many homologous and synchronized parts were identified in chromosomes A and C of *B. napus*, as well between the genome of *B. rapa* or *B. oleracea* and genome of *B. napus* (Figure 3A). In the syntenic analysis of the *Hsp90s* of *B. napus* and *A. thaliana*, five of the seven *AtHsp90s* were found to be syntenic gene pairs with 16 *BnHsp90s* (Figure 3A; Supplementary Table S4), with *AT4G24190* having the highest number of syntenic genes in *B. napus*, 5 *BnHsp90* genes, followed by *AT3G07770*, containing three syntenic *BnHsp90* genes (Supplementary Table S4; Figure 1). This indicates an expansion of the *BnHsp90* gene family in *B. napus* after its evolutionary separation from *A. thaliana*. Of all the syntenic *BnHsp90* genes, some members (*BnC02.Hsp90.5*, *BnC07.Hsp90.1*, *BnA03.Hsp90.6*, *BnC01.Hsp90.1*, *BnC08.Hsp90*) have syntenic genes in *A. thaliana*, but do not have any corresponding syntenic genes in *B. rapa* and *B. oleracea*, and three genes (*BnC09.Hsp90.2*, *BnC03.Hsp90.4*, *BnC05.Hsp90.1*) just have corresponding collinear genes in *B. oleracea* but not in *B. rapa* (Figure 3A; Supplementary Table S3). Only one gene (*BnC07.Hsp90.2*) has collinear genes in *B. rapa* but not in *B. oleracea* (Figure 3A; Supplementary Table S3). Moreover, no syntenic genes were identified in *A. thaliana* for three *BnHsp90s*, (*BnA03.Hsp90.1*, *BnC05.Hsp90.1*, *BnA03.Hsp90.6*), however, the respective collinear genes for them can be detected in *B. rapa* and/or *B. oleracea* (Figure 3A; Supplementary Table S3).

Different gene duplication may be one of the reasons for the expansion of plant gene family. Three duplication models were detected in 35 BnHsp90 genes, including whole genome duplication (WGD), dispersed and proximal. Among them, the most detected duplication mode is WGD, and there are 25 (71.4%) genes produced by WGD, followed by dispersed and proximal, both producing 5 *BnHsp90* genes (Supplementary Table S2).

### Cis-Element Analysis in the Promoters of *BnHsp90* Genes

**Figure 4:**
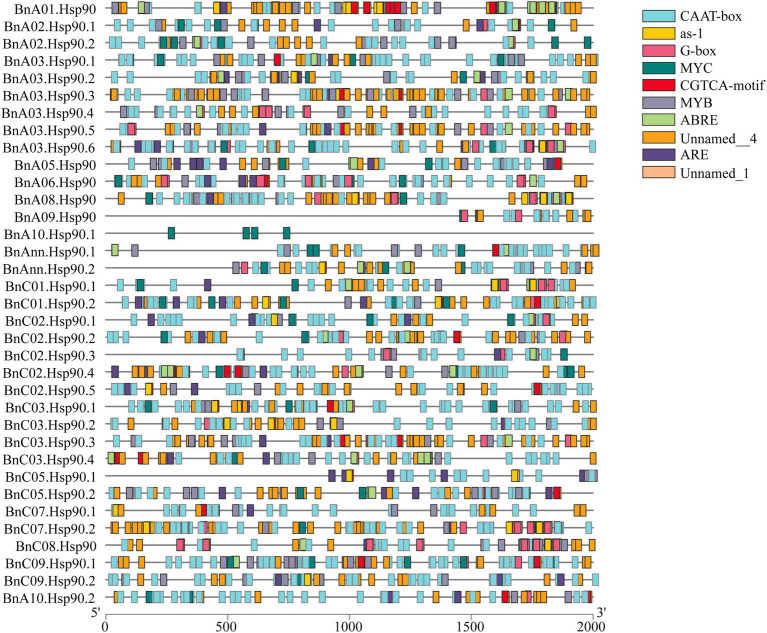
Predicted stress-related cis-elements in *BnHsp90* promoters. Promoter sequences (2,000 bp upstream region) of 35 *BnHsp90* genes were analyzed online at PlantCARE web server. Different colors were used for indicating different cis-elements, as shown on the right part.

The cis-acting elements in the promoter region play important roles in the plant responses to stress, and as well, participate in the responses to drought, ABA and other stresses (Yazaki and Kikuchi, 2005; Nakashima and Yamaguchi-Shinozaki, 2013). The ten most abundant cis-acting elements detected in *BnHsp90s* are CAAT-box, G-box, MYB, ABRE, MYC, ARE, as-1, CGTCA-motif, Unnamed_1, and Unnamed_4. CAAT-box and Unnamed_4 have the largest number of cis-acting elements identified in the *BnHsp90* promoter region, followed by G-box, MYB, ABRE and MYC. Among them, a total of 143 MYB binding sites and 93 MYC binding sites were identified in the promoter region of the *BnHsp90* genes, and 131 ABREs were identified, participating in the ABA response (Figure 4).

### Expression Analysis of *BnHsp90s* in Response to the Treatment of Salt

**Figure 5:**
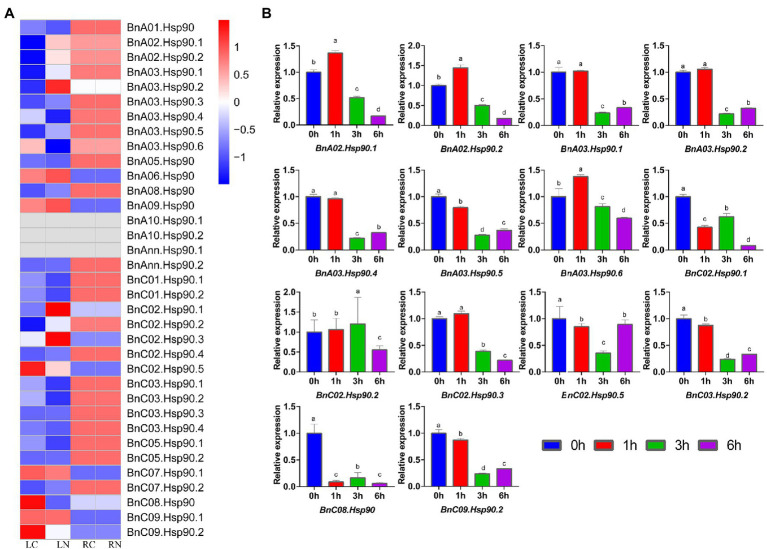
Expression changes of *BnHsp90* genes in Brassica napus after treatment with salt. **(A)** Differential expression of *Hsp90* gene in rapeseed after salt stress from public dataset (SRA datasets, PRJNA561674). The variety used in this research is Zhongshuang No. 11. LC, leaf control group; LN, leaf treatment group; RC, root control group; RN, root treatment group. **(B)** Expression patterns of *BnHsp90s* in response to NaCl treatments. Transcript levels of *BnHsp90s* were analyzed by quantitative real-time PCR using actin gene as an internal control. The unstressed expression level (0 h) was regarded as a standard. Values are the mean ± SE, *n* = 3.

In the long-term evolutionary process, plants have formed a set of response mechanisms to abiotic stresses. It is known that *Hsp90* genes will be induced in response to these abiotic stresses (Krishna, 1998). In this study, we first analyzed the transcriptome data of *B. napus* seedlings under salt stress in existing studies, and obtained the changes in the expression of *BnHsp90* gene after salt stress (Tan et al., 2019; Figure 5). After salt stress, the expression of 9 *BnHsp90* genes in *B. napus* seedling leaves was down-regulated, and the expression of 7 *BnHsp90* genes was up-regulated (Figure 5A). Among them, the expression levels of *BnA02.Hsp90.1*, *BnA02.Hsp90.2*, *BnA03.Hsp90.2*, *BnC02.Hsp90.1*, and *BnC02.Hsp90.3* changed significantly (Figure 5A). In *B. napus* roots, the changes in *BnHsp90* gene expression were less affected by salt stress (Figure 5A). We selected 14 *BnHsp90* genes with obvious changes in expression for verification (Figure 5B). In order to verify the expression changes of *BnHsp90* gene under salt stress, *B. napus* seedlings were treated with 150 mM NaCl, and seedlings of 0 h, 1 h, 3 h, 6 h post-treatment were sampled for performing qPCR on 14 *BnHsp90* genes, respectively. Following NaCl treatment, the expression of four *BnHsp90* genes (*BnA02.Hsp90.1*, *BnA02.Hsp90.2*, *BnA03.Hsp90.6*, *BnC02.Hsp90.2*,) increased first and then decreased (Figure 5B). Transcripts of ten *BnHsp90* genes (*BnA03.Hsp90.1*, *BnA03.Hsp90.2*, *BnA03.Hsp90.4*, *BnA03.Hsp90.5*, *BnC02.Hsp90.1*, *BnC02.Hsp90.3*, *BnC02.Hsp90.5*, *BnC03.Hsp90.2*, *BnC08.Hsp90*, *BnC09.Hsp90.2*) reduced after salt stress treatment (Figure 5B). Overall, more *BnHsp90* genes were reduced by the salt treatment.

### Transcriptional Changes of *BnHsp90* Genes During the Infection of *S. sclerotiorum*

**Figure 6:**
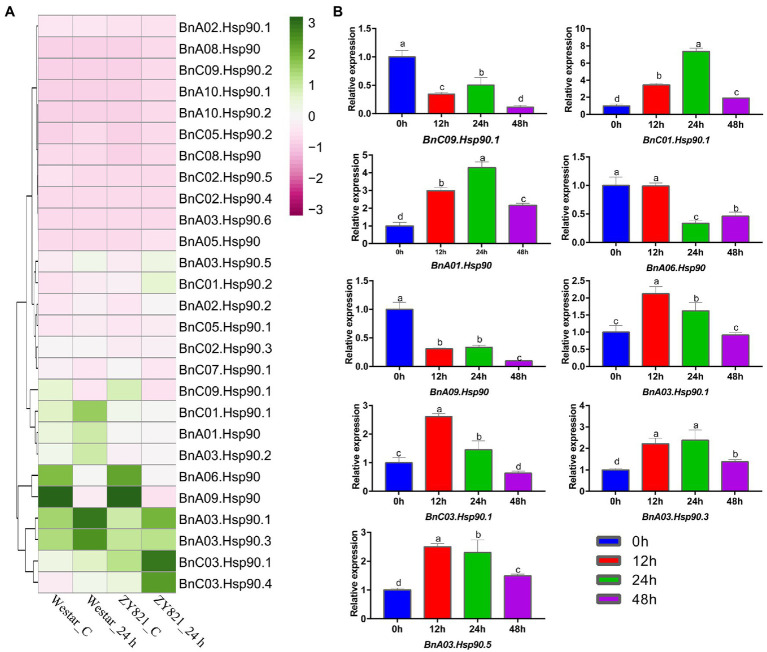
Expression of *BnHsp90* genes in *Brassica napus* infected by *Sclerotinia slcerotiorum*. **(A)** Expression changes of *BnHsp90* genes after *Sclerotinia sclerotiorum* infection from public dataset (GEO RNA-seq datasets, GSE81545). The materials used are Westar and ZY821. The treatments are divided into 0 h and 24 h post inoculation. **(B)** Expression patterns of *BnHsp90s* in response to *Sclerotinia sclerotiorum*. Transcript levels of *BnHsp90s* were analyzed by quantitative real-time PCR using actin gene as an internal control. The non-inoculated expression level (0 h) was regarded as a standard. Values are the mean ± SE, *n* = 3.

*S. sclerotiorum* is a severe pathogen on *B. napus* (Bolton et al., 2006). Previous studies have shown that the *BnHsp90* gene responds to *S. sclerotiorum* (Cao et al., 2016). In the present study, expression of *BnHsp90s* were determined according to the public RNA-seq data of *B. napus* after treatment with *S. sclerotiorum* (Girard et al., 2017). As shown in Figure 6, only 27 of the 35 *BnHsps* displayed obvious expression in the four tissues; leaves of Westar without *S. sclerotiorum* inoculation (Westar_C), Westar 24 h post-inoculated with *S. sclerotiorum* (Westar_24 h), ZY821 without *S. sclerotiorum* treatment (ZY821_C), and ZY821 24 h post-inoculated with *S. sclerotiorum* (ZY821_24 h). Nine of these 27 genes were induced for their expression by the infection of *S. sclerotiorum* in leaves of both varieties, susceptible Westar and resistant ZY821 (Figure 6A). To validate these results, nine genes were selected for qPCR verification (Figure 6B). According to the results, the expression patterns of them were coincident with the RNA-seq data (Figure 6B). There are six *BnHsp90* genes up-regulated (*BnC01.Hsp90.1*, *BnA01.Hsp90*, *BnA03.Hsp90.1*, *BnC03.Hsp90.1*, *BnA03.Hsp90.3*, *BnA03.Hsp90.5*; Figure 6B). Among them, the transcript level of *BnC01.Hsp90.1* showed the most drastic changes (Figure 6B). Three *BnHsp90* genes (*BnC09.Hsp90.1*, *BnA06.Hsp90*, *BnA09.Hsp90*) were down-regulated post inoculation by *S. sclerotiorum* (Figure 6B). These results indicate that *BnHsp90* gene may be involved in the responses of *B. napus* to sclerotinia disease. These up-regulated genes will be our target for investigating their roles in the resistance of *B. napus* to *S. sclerotiorum*.

### Subcellular Localization and Transcriptional Activity of BnA03.Hsp90.2

There are three BnHsp90 proteins (BnA03.Hsp90.2, BnA03.Hsp90.4, and BnC03.Hsp90.2) targeted to nucleus in the prediction of subcellular location. To verification of the subcellular location, one of them, BnA03.Hsp90.2 was fused with GFP to construct 103-BnA03.Hsp90.2 plasmid, which was used for transiently expressed in tobacco leaves. GFP signal was detected only in the nucleus, indicating by the mCherry signal of H2B, a nucleus-localized protein (Supplementary Figure S2A). While the GFP fluorescence (control) was observed throughout the cell (Supplementary Figure S2B). To test whether BnA03.Hsp90.2 has transcriptional activity, full length of BnA03.Hsp90.2 coding region were cloned into the pGBKT7 vector, then transformed into yeast strain AH109. Yeast cells harboring pGBKT7-BnA03.Hsp90.2, pGBKT7 (negative control) and pGBKT7-AtDREB (positive control) grew well on the SD/−Trp medium, however, only the positive control survived on the selective medium SD/−Trp-His (Supplementary Figure S2C). These results demonstrated that BnA03.Hsp90.2 was a nucleus-localized protein but did not have transactivation activity for its full-length protein in yeast.

## Discussion

With climate change, the effects of hot weather on crop growth are becoming increasingly severe, and studying the responses of plants to high temperature stress is becoming increasingly important for plant growth (Suzuki et al., 2014). When the temperature increases 5°C compared to the normal temperature, the physiological and biochemical reactions in the organism are inhibited, and most of the protein and mRNA transcription are inhibited by heat stress (Mittler et al., 2012; Raja et al., 2020). High temperature stress usually alters the expression of stress-related genes in the plant, and there is an important type of protein heat shock proteins, Hsp, which is rapidly synthesized after the heat stress treatment (Pearl and Prodromou, 2000), to cope with the rise in temperature (Tissières et al., 1974). Heat shock protein 90 has been identified in many plants; however, little is known about Hsp90 in rapeseed. Here, we conducted a comprehensive identification and genetic analysis of the rape *Hsp90* gene family, as well as expression analysis of them in response to salt and pathogen infection, which sheds light on their possible functions in *B. napus*.

Compared with the *Hsp90* gene family identified in other plants (7 in *A. thaliana* and 9 in *rice*; Krishna and Gloor, 2001), a total of 35 *Hsp90* genes were identified from *B. napus*, suggesting a large increase in the gene number. This may be due to polyploidization of the *B. napus* (Chalhoub et al., 2014). Seventeen and 18 *Hsp90* genes were obtained from *B. rapa* and *B. oleracea*, respectively, in the present study, and the total number of them is identical to the number of *Hsp90* genes in *B. napus*, which is in accordance with the fact that the allotetraploid *B. napus* is formed by the hybridization of *B. rapa* and *B. oleracea* (Snowdon et al., 2002; Rana et al., 2004; Chalhoub et al., 2014). Their phylogenetically related species *A. thaliana* has 7 *Hsp90* genes (Krishna and Gloor, 2001), which is around 1/3 of the number of *Hsp90s* in *B. rapa* or *B. oleracea*, and may be explained by the whole genome triplication (WGT) of *B. rapa* and *B. oleracea* after their split with *A. thaliana* as well as the extensive block reshuffling and chromosome reduction following WGT (Mun et al., 2009; Cheng et al., 2014). As the ‘A’ and ‘C’ genome of *B. napus* originates from *B. rapa* and *B. oleracea*, respectively (Chalhoub et al., 2014), the WGT in the chromosomes of *B. rapa* and *B. oleracea* could explain that the main duplication type of *BnHsp90s* is WGD (71.4%). Collinearity analysis have shown that *B. rapa* and *B. oleracea* experienced extensive gene loss compared with *A. thaliana* following triplication during evolution (Town et al., 2006; Mun et al., 2009). We found that some *BnHsp90* genes lost their homologues in *B. rapa* or/and *B. oleracea*, which revealed that *B. rapa* and *B. oleracea* underwent gene loss after evolutionary split from the ancestors for forming *B. napus*. In addition, some *BnHsp90s* missed respective homologs in *A. thaliana*, providing an example of extensive genome fractionation and block reshuffling after triplication in *Brassica* (Cheng et al., 2014). In summary, the *BnHsp90* genes in *B. napus* may be mainly produced by gene duplication followed by gene loss occurring during the evolution process.

Duplication and divergence have been proposed to be the major force to drive the evolution of new genes as well as an increase in the gene diversity (Meyer and Schartl, 1999; Taylor and Raes, 2004; Magadum et al., 2013). The phylogenetic analysis of *Hsp90* genes generated five groups based on *Hsp90s* of *B. napus*, *B. rapa*, *B. oleracea*, and *A. thaliana*, with no *A. thaliana Hsp90* gene from group IV, suggesting a high divergence of the *Hsp90* gene family during the evolution of *Brassica* plants. Almost all the genes in group I, II, and III were predicted to be located in the mitochondria, which also indicated that evolutionarily related *Hsp90* genes appeared in similar subcellular locations. Three BnHsp90 proteins (BnA03.Hsp90.2, BnA03.Hsp90.4, and BnC03.Hsp90.2) were predicted to be localized to the nucleus, and BnA03.Hsp90.2 was validated to locate in the nucleus, which is in accordance with its evolutionary related Arabidopsis protein HSP90.2 (AT5G56030; Watanabe et al., 2016). BnA03.Hsp90.2 does not have transactivation activity in yeast cells, suggesting that it may not function as a transcription factor. Another possibility is that the full-length protein of BnA03.Hsp90.2 does not have transactivation activity or needs additional modification to enable its transactivation.

Intron gain and/or intron loss, and intron densities were proposed to play an important role in the evolution of large eukaryote genomes (Jeffares et al., 2006). In the present study, the analysis of the gene structure of different groups indicated that the number of exons and exon-intron structures of the *Hsp90* genes in each group were very close. The identified 5 motifs were highly conserved across different BnHsp90s, and were discovered to be consistent with previous studies on *Brachypodium distachyon* (L.) P. Beauv. *Hsp90* gene (Zhang et al., 2017). Also, the distribution of these motifs in the same group shows a similar pattern. These analyses suggested that the evolutionary classification of *BnHsp90* genes is reliable.

Salt stress is one of the most severe environmental factors that restrict crop production around the world (Yokoi et al., 2002), and *B. napus* is sensitive to salt stress, which inhibits their growth and production markedly (Pace and Benincasa, 2010). In this study, some of the *Hsp90* gene family members were found to be induced by the salt treatment, such as *BnA03.Hsp90.2*, *BnA06.Hsp90*, *BnA09.Hsp90*, *BnC02.Hsp90.1*, and *BnC02.Hsp90.3*. *BnA03.Hsp90.2, BnC02.Hsp90.1, and BnC02.Hsp90.3* were classified into a group without a homologous gene from *A. thaliana*, indicating that *BnA03.Hsp90.2, BnC02.Hsp90.1*, and *BnC02.Hsp90.3* may be evolutionarily distant from their ancestor in *A. thaliana* and may be involved in a different mechanism for salt responses. The closely related gene *AT2G04030* of *BnA06.Hsp90* and *BnA09.Hsp90* was found to enhance plant sensitivity to salt (Song et al., 2009), suggesting that *BnA06.Hsp90* and *BnA09.Hsp90* may not only be induced by salt but also act as a negative modulator in response to salt treatment. In addition, the promoter region was analyzed, and a large number of cis-acting elements involved in abiotic stress and hormone regulation were predicted in these genes. For example, a total of 143 MYB binding sites and 131 ABRE binding sites in the ABA regulatory pathway were predicted in the promoters of these genes. MYB transcription factors and ABA-regulated genes participate in a variety of biotic and abiotic stress responses of plants (Ambawat et al., 2013; Nakashima and Yamaguchi-Shinozaki, 2013). However, the induction of *BnHsp90s* by salt is not so consistent between our qPCR results, for example, the expression of *BnA03.Hsp90.2*, *BnA03.Hsp90.5*, *BnA03.Hsp90.6*, *BnC02.Hsp90.1*, and *BnC02.Hsp90.3*, and the published RNA-seq data. This difference might be due to the whole seedlings used in the present test but not the separate root and leaf tissues as determined by Tan et al. (2019), as well as the time points tissues sampled after treatment being different.

*Hsp90* genes have been reported to be related to disease resistance (Hubert et al., 2003; Kanzaki et al., 2003; Takahashi et al., 2003), and here we also found that expression of six *BnHsp90s* (*BnC01.Hsp90.1*, *BnA01.Hsp90*, *BnA03.Hsp90.1*, *BnA03.Hsp90.3*, *BnC03.Hsp90.1*, and *BnA03.Hsp90.5*) is elicited by the infection of *S. sclerotiorum*, which caused Sclerotinia stem rot, one of the most important diseases on oilseed rape. There are 5 orthologs of *AT4G24190*, but only two of them, *BnC01.Hsp90.1* and *BnA01.Hsp90*, are induced by the infection of *S. sclerotiorum*, which revealed the functional divergence of paralogs (Soria et al., 2014). Furthermore, the other two induced genes by *S. sclerotiorum*, *BnA03.Hsp90.3* and *BnA03.Hsp90.5*, were two evolutionarily closely related genes, indicating a conserved mechanism in response to *S. sclerotiorum* by *BnHsp90* genes.

*BnA03.Hsp90.2* is evolutionarily related with the three Arabidopsis *Hsp90* genes, *AT5G56030* (*AtHsp90.2*), *AtHsp90.3* (*AT5G56010*), and *AtHsp90.4* (*AT5G56030*). And it was confirmed to be localized in the nucleus in the present study, as its close protein AtHsp90.2 (Clément et al., 2011). AtHsp90.2 and AtHsp90.3 are critical for maintaining appropriate levels of immune receptor proteins (Huang et al., 2014), in addition, these two proteins regulate RPP4-mediated temperature-dependent cell death and defense responses (Bao et al., 2014). Expression of *BnA03.Hsp90.2* was up-regulated by the infection of *S. sclerotiorum* in the susceptible variety Westar, which suggests that it may also participate as its homologous genes in *Arabidopsis*, and it will be of interest to investigate its role against *S. slcerotiorum* in *B. napus*.

## Conclusion

Thirty-five *BnHsp90s* were identified through the whole genome of *Brassica napus* and were divided into 5 groups. BnHsp gene family was suggested to be shaped by whole gene duplication followed by gene losses during evolution process. Expression of five *BnHsp90* genes, *BnA02.Hsp.1*, *BnA02.Hsp.2*, *BnA03.Hsp.6*, and *BnC02.Hsp.2* were induced by the treatment of salt. And nine genes including *BnA03.Hsp.1*, *BnA03.Hsp.2*, *BnA03.Hsp.4*, *BnA03.Hsp.5*, *BnC02.Hsp.1*, *BnC02.Hsp.3*, *BnC03.Hsp.2*, *BnC08.Hsp.,* and *BnC09.Hsp.2* were revealed to decrease their transcription following the salt stress treatment. Six genes including *BnC01.Hsp90.1*, *BnA01.Hsp90*, *BnA03.Hsp90.1*, *BnC03.Hsp90.1*, *BnA03.Hsp90.3*, *BnA05.Hsp90.5*, were validated to be up-regulated by the infection of *S. sclerotiorum* in *B. napus*, and three genes (*BnC09.Hsp90.1*, *BnA06.Hsp90*, *BnA09.Hsp90*) were confirmed to be down-regulated upon the treatment of *S. sclerotiorum*. These salt- or *S. sclerotiorum* -responsive *BnHsp90* genes will be our targets for investigating their roles in the defense responses of *B. napus* against salt or *S. sclerotiorum*.

## Data Availability Statement

The datasets presented in this study can be found in online repositories. The names of the repository/repositories and accession number(s) can be found in the article/Supplementary Material.

## Author Contributions

FL, DH, and DW conceived and designed the experiments. FL contributed to the bioinformatic analysis. LW, LJ, and BX performed the experiments. BX prepared the samples for RNA extraction. LW and LJ conducted the RNA extraction and qPCR analysis. LW analyzed the data. LW and FL wrote the manuscript. FL and YW reviewed the manuscript. All authors contributed to the article and approved the submitted version.

## Funding

This research was partly supported by the project of the ability establishment of sustainable use for valuable Chinese Medicine Resources (2060302), Science and Technology Project of Henan Province (202102110156), Natural Science Foundation of Henan Province (202300410151), and the Key Scientific Research Projects in Colleges and Universities of Henan Province (22A210011).

## Conflict of Interest

The authors declare that the research was conducted in the absence of any commercial or financial relationships that could be construed as a potential conflict of interest.

## Publisher’s Note

All claims expressed in this article are solely those of the authors and do not necessarily represent those of their affiliated organizations, or those of the publisher, the editors and the reviewers. Any product that may be evaluated in this article, or claim that may be made by its manufacturer, is not guaranteed or endorsed by the publisher.

## Footnotes

^1^ http://www.arabidopsis.org/

^2^ https://www.genoscope.cns.fr/brassicanapus/

^3^ http://brassicadb.cn/

^4^ https://meme-suite.org/

^5^ http://bioinformatics.psb.ugent.be/webtools/plantcare/html/
