# Supplementary figures and images for: Genome Structures and Evolution Analysis of Hsp90 Gene Family in Brassica napus Reveal the Possible Roles of Members in Response to Salt Stress and the Infection of Sclerotinia sclerotiorum
Source: Front Plant Sci. 2022 Apr 7;13:854034. doi: 10.3389/fpls.2022.854034 (PMC9022010; doi:10.3389/fpls.2022.854034)

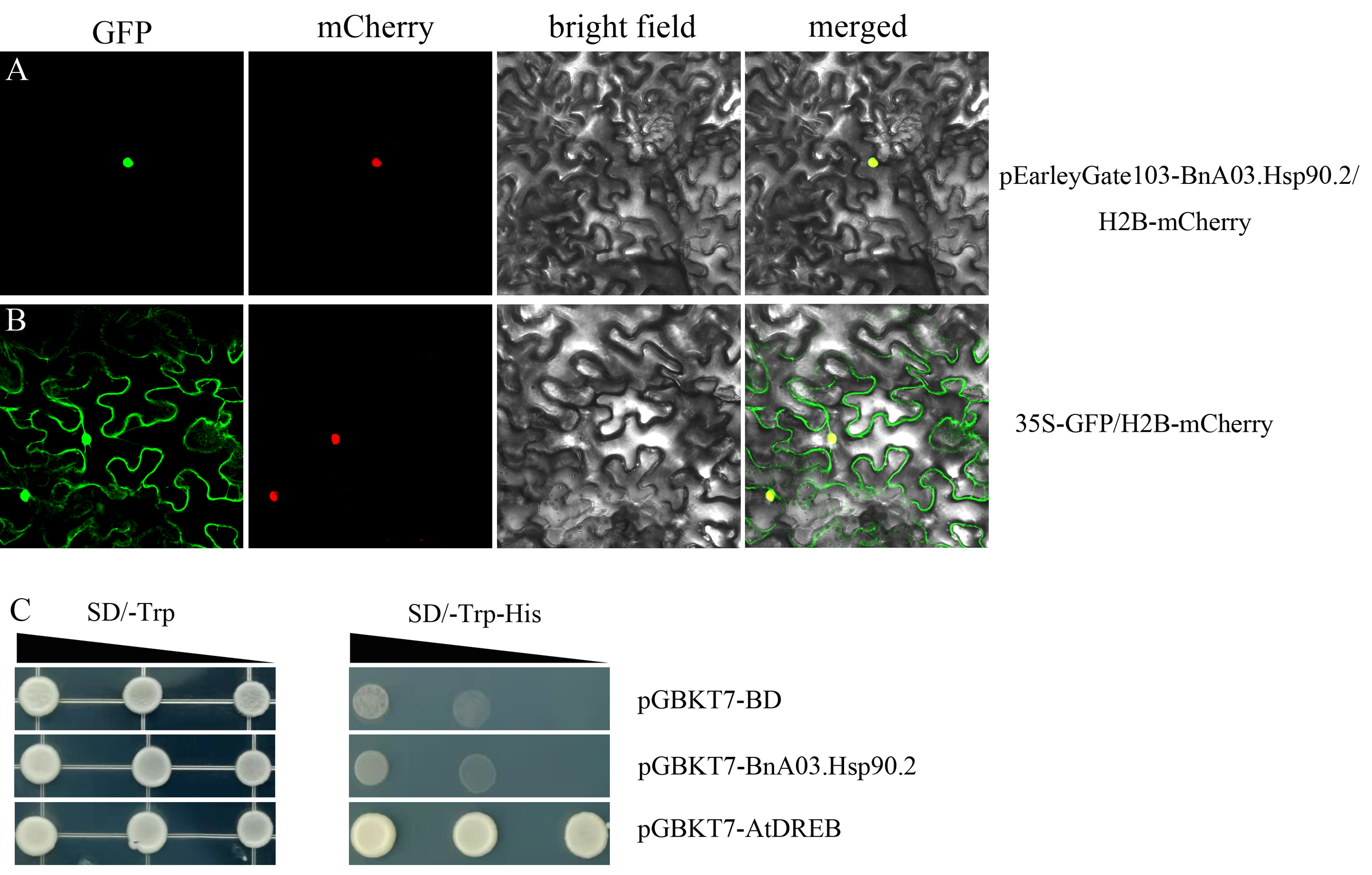

Supplement: Supplementary file 1 [file Data_Sheet_1.ZIP › Figure S2.tif]

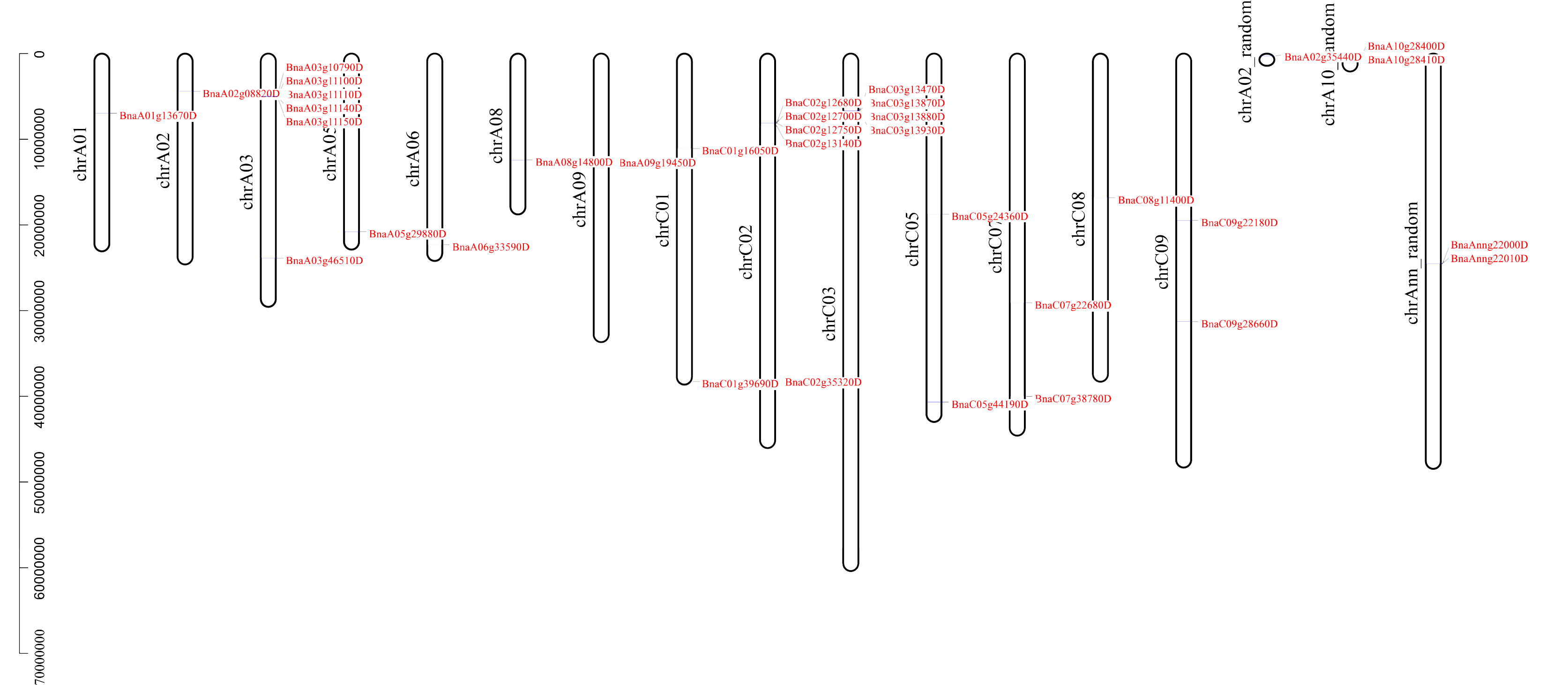

Supplement: Supplementary file 1 [file Data_Sheet_1.ZIP › Figure S1.tif]
